# Exosome and epithelial–mesenchymal transition: A complex secret of cancer progression

**DOI:** 10.1111/jcmm.17755

**Published:** 2023-05-15

**Authors:** Rajib Dhar, Arikketh Devi, Sukhamoy Gorai, Saurabh Kumar Jha, Athanasios Alexiou, Marios Papadakis

**Affiliations:** ^1^ Cancer and Stem Cell Biology Laboratory, Department of Genetic Engineering SRM Institute of Science and Technology Kattankulathur India; ^2^ Rush University Medical Center Chicago Illinois USA; ^3^ Department of Biotechnology, School of Engineering and Technology (SET) Sharda University Greater Noida India; ^4^ Department of Biotechnology Engineering and Food Technology Chandigarh University Mohali India; ^5^ Department of Biotechnology, School of Applied and Life Sciences (SALS) Uttaranchal University Dehradun India; ^6^ Department of Science and Engineering, Novel Global Community Educational Foundation Hebersham New South Wales Australia; ^7^ AFNP Med Wien Austria; ^8^ Department of Surgery II University Hospital Witten‐Herdecke Witten Germany

**Keywords:** anti‐metastasis therapeutic development, cancer, epithelial to mesenchymal transition, metastasis, organ‐specific metastasis

## Abstract

This short communication will enlighten the readers about the exosome and the epithelial‐mesenchymal transition (EMT) related to several complicated events. It also highlighted the therapeutic potential of exosomes against EMT. Exosome toxicology, exosome heterogeneity, and a single exosome profiling approach are also covered in this article. In the future, exosomes could help us get closer to cancer vaccine and precision oncology.

## 1 BACKGROUND

Cancer is the most challenging health crisis worldwide. In this disease, the cells lose control of cellular proliferation phase as a result, this alteration leads to tumour development. On the next level, tumour development is associated with multiple phases, leading to more complex health complications. The tumour is the fundamental block of cancer development. There a group of cellular reprogramming takes place. Metastasis is one of them. In metastasis, cells lose adhesion properties, migrate to distant organs, and develop a secondary tumour. The detailed exploitation of metastasis mentions that epithelial–mesenchymal transition (EMT) is the major event that plays a crucial role in cancer metastasis. In this process, the modulation of the extracellular matrix (ECM) is also a vital event, and exosome‐associated fibronectin influences it.^1^ In the current decade, scientific research is unraveling a secret connection related to metastasis and exosomes.^2^ The exosome is the subpopulation of extracellular vesicles (EVs) and it originates from endosomes. In general, it is related to cellular communication and carries the status of parental cells (Health or pathological condition). Exosomes based molecular transport develops the significant impact in diagnostic and prognostic‐related cancer biomarker research. An exciting fact about exosomes is that several pieces of scientific evidence worldwide highlight that exosomes are strongly involved in cancer metastasis. Tumour‐derived exosomes (TEXs) associated molecular cargos (DNA, RNA, proteins, and lipids) are actively influenced by cancer metastasis. TEXs promote angiogenesis, immunomodulation, and metastasis of cancer cells.^2^

## 2 BIOGENESIS OF EXOSOMES

Exosomes originate from the endosomes, and exosome biogenesis is based on two different pathways, dependent on the endosomal sorting complex required for transport (ESCRT) and independent of the ESCRT complex. ESCRT complex constructs with four significant components ESCRT‐0, ESCRT‐I, ESCRT‐II, and ESCRT‐III.^2^ Exosomes biogenesis is initiated via endosome formation. An early endosome is converted into a late endosome when it is full of intraluminal vesicles (ILVs). ILVs mature in the multivesicular bodies (MVBs). MVBs carrying ILVs later mature in the exosome. ESCRT‐0 binds with a ubiquitinated side of the biomolecules and ESCRT‐I is activated via ESCRT‐0 after ESCRT‐I and ESCRT‐II are involved in the cytoplasmic cargo selection process and guidance of ESCRT‐0. ESCRT‐III plays a vital role in the exosome budding process. ESCRT‐independent pathway^2^ leads via exosome surface tetraspanin proteins, lipids, and ceramide. In this process, lipids (sphingomyelin, cholesterol) and ceramide are involved in membrane budding, and tetraspanin proteins (CD63, CD81, CD9, etc.) is associated with exosome formation.

## 3 IMMUNE MODULATION

Immune cells modulation is one of the vital hallmarks of cancer. In this stage, TEXs associated with several molecules alter the antitumor function of immune cells and promote cancer progression. Myeloid‐derived suppression cells (MDSCs) are immature population of myelod cells, they are suppress immune system during cancer. TEXs regulated MDSCs involvement in carcinogenesis via miRNA and heat shock proteins.^3^ Macrophages are groups of cells related to antigen presentation. TEXs miRNA promotes M2 polarization which enhances cancer metastasis.^2^ Dendritic cells (DCs) are called professional antigen‐presenting cells (APCs) because they express MHC‐I and MHC‐II (they can present exogenous and endogenous antigens). TEXs miRNA‐212‐3p regulate DCs mediated Immune suppression in cancer.^3^ Natural killer (NK) cells are a group of cells that are part of innate immunity. TEXs mediated NK cells NKG2D legend down expression and miRNA reduces its cytotoxicity against cancer. B cells are major players in the humoral immunity of the human body. TEXs enhance the proliferation of B regulatory (B‐reg) cells, that involved in the immune surveillance escape of cancer cells. T cells control cellular immunity in the human body. The molecular cargo of TEXs induces T cell apoptosis and suppresses the anticancer immune response. Tumour derived exosomes enhance the proliferation of regulatory T cells (T‐reg) as a result of suppress on of immune escape via TGF‐β and IL‐10. TEXs surface PDL1 and FasL regulates helper T cells (Th) associated immune suppression.^2^

## 4 BEGINNING OF METASTASIS

**FIGURE 1:**
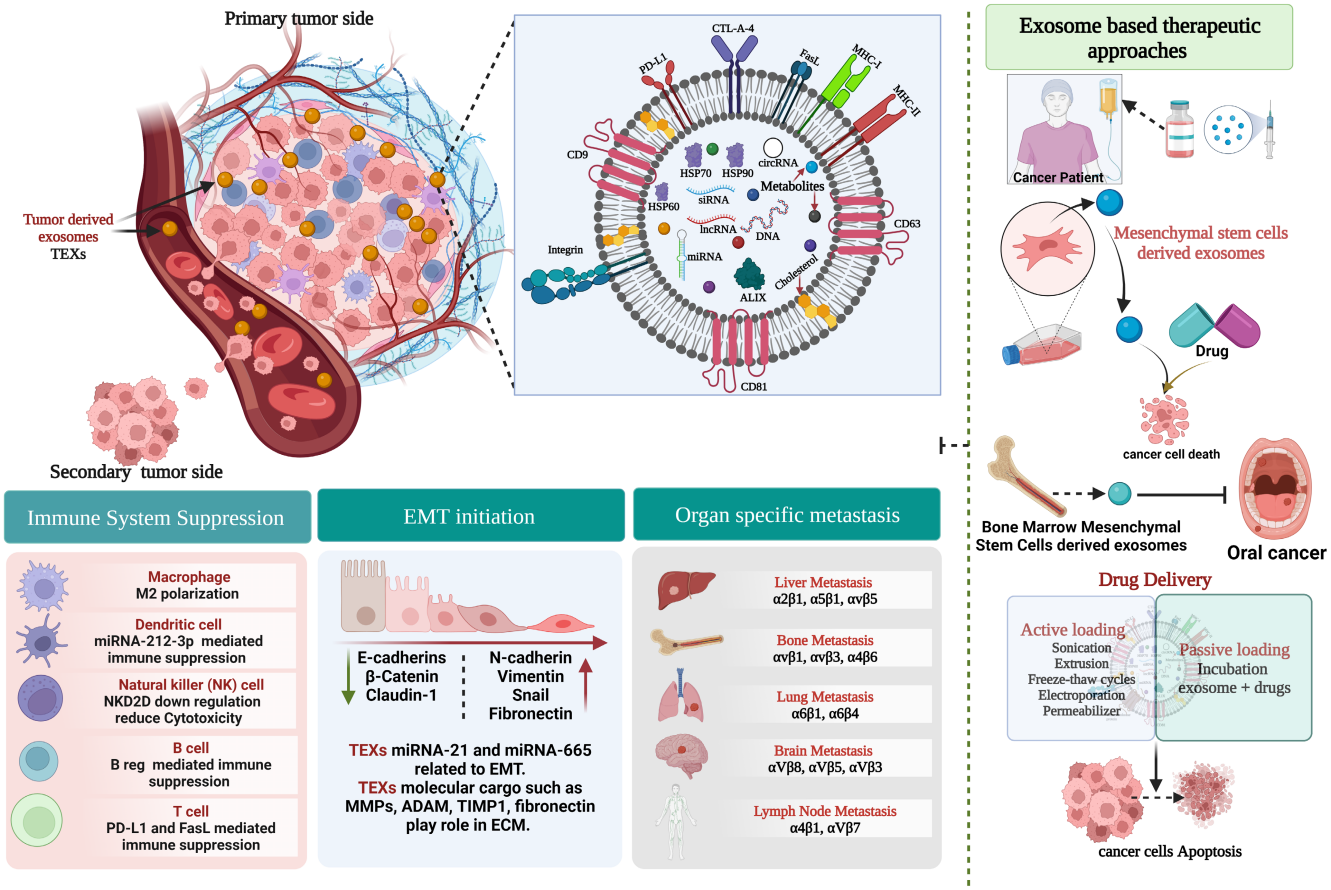
EMT and exosome interrelation and therapeutic approaches. Tumour‐derived exosomes (TEXs) are involved in immune cells reprogramming, extracellular matrix remodelling (ECM), EMT and organ‐specific metastasis. (ADAM‐A Disitegrin and metalloproteinase, TIMP‐1‐Tissue Inhibitor Metalloproteinase‐1) (Created with Biorender.com).

The cellular physical alteration in the tumour microenvironment (TME) regulates initiation of metastasis. After this process, cancer cells cross the biological barrier and enter in the circulatory system.^4^ Tumour cell mortality develops in the EMT process (Figure 1). TEXs regulate several oncogenic cellular signalling pathways. In EMT, the hippo pathway is responsible for the higher expression of the mesenchymal marker N‐cadherin and Vimentin compare to E‐cadherin. TEXs‐related miRNA‐301 activated Wnt/β‐catenin signalling pathways. TEXs associated miRNA‐21 mediated upregulation of EKT, and AKT signalling pathways promotes more hypoxic conditions and EMT initiation. TEXs‐related molecular signatures such as Metrix melano‐proteases (MMPs), tumour necrosing factor (TNF), integrin, and interleukin promote metastasis under hypoxic conditions.^5^ TEXs surface protein gives guidance to cancer cells for distance organ migration.

## 5 PREMETASTATIC NICHE FORMATION

The premetastatic niche (PMN) is the cluster of tumour cells localized before organ‐specific metastasis. In this event, exosome surface expressing protein and miRNA‐associated cellular communication promote premetastatic niche formation. Exosomes intragrain enhance inflammation. Indirectly PMN formation regulates by the high expression of tumour necrosing factor alpha (TNF‐α), vascular endothelial growth factor (VEGF) and interleukin (IL).^6^ PMN formation also develops an immune suppression mechanism based on the exosome PD‐1 legend. TEXs PD‐L1 inhibits immune cells development, alters functionality and initiates apoptosis of immune cells. Furthermore, the PMN‐secreted exosomes miRNA‐23‐3p^7^ are related with angiogenesis. Bone marrow‐derived cells (BMDCs) release exosomes express fibronectin to support PMN development.^8^ TEXs DNA (carries the massage of cancer mutation), RNAs (miRNA‐139 and miRNA‐21), long noncoding RNA (LINC00512) and circular RNA also involved a vital role in PMN formation. Exosome‐associated higher expression of CD44 and MMP proteins led to PMN formation.^9^ Finally, TEXs regulate PMN formation before and after metathesis.

## 6 ORGAN‐SPECIFIC METASTASIS

In cancer, organ‐specific metastasis is the most exciting part of EMT. Recent scientific research indicates that TEXs surface integrin supports the migration of circulating cancer cells in certain organs.^10^ Integrin of the cell membrane related to cell signalling, adhesion, extracellular matrix (ECM) remodeling and metastasis. Integrins of TEXs consists of heteromeric component α (18 types) subunit and β (8 types) subunit. Both subunits combinedly make 24 molecular signalling ligands. Tumour cells release exosomes α6β4 and αvβ3 integrins expression associated with lung metastasis.^10^ Integrins also lead to bone metastasis (αvβ6, αvβ3 and α4β1), brain metastasis (αvβ3, αvβ5 and αvβ8), liver metastasis (α2β1, αvβ5 and α5β1) and lymph node metastasis (α4β1 and α4β7).^10^, ^11^

## 7 EXOSOME‐BASED THERAPEUTIC APPROACHES IN METASTASIS INHIBITION

Exosome‐associated dynamic bioactive cargos have a potential role in cancer therapeutics, as evidence shows that exo‐miRNA‐30 reduces EMT by downregulating for SANAIL expression. miRNA‐375 suppresses EMT associated with brain, prostate, gastric and ovarian cancer. Fibroblast and mesenchymal stem cells (MSDExo) derive exosome cargo miRNA‐148 inhibition of EMT.^12^, ^13^ B cell‐derived exosomes miRNA‐335 shows an impact on anticancer activity in EMT. miRNA‐21 regulated EMT associated with several gene expressions and overcame cancer chemoresistance.^14^ Oral cancer‐associated EMT inhibits via miRNA‐101 exosomes released from bone marrow‐derived mesenchymal stem cells (BMMSCs). Exosome‐related miRNA‐34 inhibits oesophageal cancer regarding oncogenic cellular signalling. In the drug delivery process, exosomes are a more efficient tool for targeting cancer.^15^ Mesenchymal stem cells (MSCs)‐derived exosomes loaded (incubation method) Doxorubicin drug inhibits pancreatic cancer cell proliferation. Breast cancer cells–derived exosomes and transfected miRNA‐134 reduce breast cancer metastasis and prevent the development of chemoresistance. Exosomes show more promising results in an in vitro and in vivo model. Several clinical trails are going on exosome‐based therapeutic development. A combination of dendritic cell‐derived exosomes and cancer antigen clinical trail (phage I) for skin cancer and lung cancer shows low toxicity and the development of a robust immune response against both cancers. The most exciting thing is that exosomes derived from plant cells^16^ will be used for the development of cancer therapeutics (clinical trial ongoing) against colon cancer. Artificial exosomes and chimeric exosomes are the leading initiators of the new era of cancer therapeutics development.

## 8 CONCLUSION

Exosome research transforms cancer‐associated liquid biopsy. TEXs are a promising source of cancer biomarkers.^15^ TEXs regarding molecule signature in cancer, if we clearly understand that, may open a new therapeutic era for cancer. This area of research holds several complex questions, such as exosome subpopulation and exosome size, and same cancer cells derive exosomes that carry the same biological molecules or they are altered with developing cancer. Exosome research must require isolation and characterization sensitive and efficient methods. This domain of research faces the most complicated question is exosome heterogeneity (It is regulated via several factors such as exosomes origin, size and internal molecular diversity).^17^ This limitation has been overcome via a Single exosome profiling approach.^17^ This process combines several multidisciplinary ways such as nanotechnology‐based platforms (microfluidic devices, exosome sensors, Nanopore), multiomics profiling (Genomics, Transcriptomics and Proteomics), and machine learning.^17^ Single exosome profiling protocol supports us in decoding exosome heterogeneity and exosomes‐based more specific and efficient theranostic approaches development in cancer research. Also, a highlighted research domain is luminescence material development which gives a clear idea about exosome secretion and uptake mechanisms. Exosome‐associated epigenetic reprogramming needs further investigation. Toxicology and exosome field are under investigation domain. Mesenchymal stem cell‐derived exosomes are nontoxic (in vivo study).^18^ Researchers mention that after parental cells release exosomes, travelling to different parts of the human body and chemical exposure can regulate the toxic nature of exosomes.^19^ In vitro study, highlighted that exosomes cargo mediated toxicity related in several pathological complecation (cancer, neurological disease, and pulmonary disease).^20^ This analysis is based on primary cell culture and organ‐on‐a‐chip methods.^20^ Toxicology and exosome domain required more scientific investigation for clinical application. Exosome theragnostic^15^ required more detailed clinical trials for future exosome‐based vaccine^21^ development. Exosome research with all the limitations of exosomes, if kept aside, there is no confusion that exosome is a new dimension of cancer research. In the future, exosome will help us develop more promising biomarkers and introduce precision medicine for cancer.

## AUTHOR CONTRIBUTIONS

**Rajib Dhar:** Conceptualization (equal); data curation (equal); writing – original draft (equal); writing – review and editing (equal). **Arikketh Devi:** Conceptualization (equal); data curation (equal); writing – original draft (equal); writing – review and editing (equal). **Sukhamoy Gorai:** Conceptualization (equal); data curation (equal); writing – original draft (equal); writing – review and editing (equal). **Saurabh Kumar Jha:** Conceptualization (equal); data curation (equal); writing – original draft (equal); writing – review and editing (equal). **Athanasios Alexiou:** Writing – original draft (equal); writing – review and editing (equal). **Marios Papadakis:** Writing – original draft (equal); writing – review and editing (equal).

## ACKNOWLEDGMENTS

Open Access funding enabled and organized by Projekt DEAL.

## FUNDING INFORMATION

There is no funding for this study.

## CONFLICT OF INTEREST STATEMENT

The authors confirm that there are no conflicts of interest.

## Data Availability

Data sharing is not applicable to this article as no new data were created or analysed in this study.
